# Cardiovascular risk factors and acute-phase response in idiopathic ascending aortitis: a case control study

**DOI:** 10.1186/ar2633

**Published:** 2009-02-27

**Authors:** Vaidehi R Chowdhary, Cynthia S Crowson, Kimberly P Liang, Clement J Michet, Dylan V Miller, Kenneth J Warrington, Eric L Matteson

**Affiliations:** 1 Division of Rheumatology, Department of Medicine, Mayo Clinic College of Medicine, 200 1st Street SW, Rochester, MN 55905, USA; 2 Division of Biostatistics, Department of Health Sciences Research, Mayo Clinic College of Medicine, 200 1st Street SW, Rochester, MN 55905, USA; 3 Department of Medicine and Division of Rheumatology, University of Pittsburgh Medical Center, 200 Lothrop Street, Pittsburgh, PA 15213, USA; 4 Division of Anatomic Pathology, Department of Laboratory Medicine and Pathology, Mayo Clinic College of Medicine, 200 1st Street SW, Rochester, MN 55905, USA

## Abstract

**Introduction:**

Idiopathic aortitis is a rare condition characterized by giant cell or lymphoplasmacytic inflammation of the aorta. The purpose of this study was to describe risk factors for the development of idiopathic aortitis.

**Methods:**

We conducted a case control study of 50 patients who were age-matched with two control subjects with non-inflammatory ascending aortic aneurysms. We examined whether the prevalences of gender, hypertension, hyperlipidemia, diabetes mellitus, smoking, family history of any aortic aneurysms, and elevated inflammatory markers differed between cases and controls.

**Results:**

The mean age of cases was 71.6 ± 8.9 years and that of controls was 71.1 ± 8.9 years. We found female gender (odds ratio [OR] 2.41, 95% confidence interval [CI] 1.20 to 4.85; *P *= 0.014) and active smoking (OR 3.37, 95% CI 1.12 to 10.08; *P *= 0.03) to be associated with idiopathic aortitis. The association with smoking persisted after adjustment for gender (OR 3.24, 95% CI 1.05 to 9.96; *P *= 0.04). There was a trend toward lower prevalence of diabetes mellitus in cases (OR 0.39, 95% CI 0.11 to 1.43; *P *= 0.16) but no difference in prevalences of other risk factors. The median pre-operative erythrocyte sedimentation rate (ESR) was 20 mm/hour in cases (n = 13) and 9 mm/hour in controls (n = 22). The median pre-operative C-reactive protein (CRP) levels were 12 mg/L in cases (n = 8) and 3 mg/L in controls (n = 6) (normal: <8 mg/L). A higher proportion of cases versus controls had elevations in ESR (38% versus 9%; *P *= 0.075) and CRP (62% versus 0%; *P *= 0.031).

**Conclusions:**

Gender and smoking may interact in complex mechanisms with immune and proteolytic pathways in older, less distensible thoracic aortas. Elevated acute-phase reactants as a marker of systemic inflammation may be present in some patients.

## Introduction

Aneurysms of the thoracic aorta are rare and occur with an incidence of 5.9 per 100,000 [1]. They are caused by weakening of the aortic wall from hypertension, heritable disorders like Marfan syndrome, bicuspid valve disease, and inflammatory and infectious processes. Among the systemic inflammatory diseases, thoracic aneurysms and aortitis occur in giant cell arteritis (GCA), Takayasu arteritis, anti-neutrophil cytoplasmic antibody (ANCA)-associated granulomatous vasculitis, spondyloarthropathies, rheumatoid arthritis, and systemic lupus erythematosus [2]. Rarely, a primary inflammatory process characterized by lymphoplasmacytic or giant cell infiltrate may be responsible. Such patients are termed to have idiopathic or isolated aortitis, a condition that is likely different from GCA or temporal arteritis.

The risk factors for development of aortic complications in GCA have been well studied [3]. The presence of an aortic insufficiency murmur, hypertension, coronary artery disease, hyperlipidemia, and symptoms of polymyalgia rheumatica (PMR) and elevation of systemic markers of inflammation were predictors of aneurysm development [4,5]. Persistent untreated inflammation was postulated as one mechanism for weakening of aortic wall and consequent aortic complications. A prospective study of 54 GCA patients who were screened with a defined protocol found aortic aneurysms in 22% of patients [6]. Aneurysms were more common in men and occurred less frequently in patients with hypercholesterolemia. Treatment of hypercholesterolemia with statins was postulated to be protective for aortic wall enlargement. There was no difference in the prevalences of smoking, hypertension, and diabetes in patients with or without aortic abnormalities. Interestingly, at the time of screening, patients with aneurysm/aortic dilatation had lower serum acute-phase reactants and lower relapse rates and needed shorter periods of prednisone therapy [6].

Histopathologic examination of the aortic wall revealed a paucity of inflammatory infiltrate but multiple foci of disruption of elastic lamellae, even in areas devoid of inflammation. There was increased expression of matrix metalloproteinase (MMP)-2 in the temporal artery as well as aortic tissue, whereas MMP-9 was found only in temporal artery specimens with active inflammation [6]. Thus, the process of aneurysm formation in systemic inflammatory diseases is complex, multifactorial, and likely involves immune and proteolytic pathways.

The risk factors for development of idiopathic aortitis are not known. The problem is compounded by the fact that many patients are diagnosed post-operatively after histopathologic review of the surgical specimen reveals giant cell inflammation. Information on pre-operative traditional markers of inflammation such as erythrocyte sedimentation rate (ESR) and C-reactive protein (CRP) is scanty [7-9]. In this case control study, we examined whether traditional cardiovascular risk factors differed in patients with idiopathic aortitis compared with patients with non-inflammatory aneurysms. We also assessed the frequency of abnormal pre-operative ESR and CRP in these patients.

## Materials and methods

### Subjects

Medical records of all patients at least 18 years old who underwent surgical resection for ascending aortic aneurysm from 1 January 2000 until 31 July 2006 were searched by means of a database of pathology specimens. Patients with giant cell or lymphoplasmacytic aortic inflammation were identified, and the histopathology slides were reviewed (DVM). Individuals with aortitis due to identifiable systemic rheumatic diseases, infectious diseases, and heritable diseases (Marfan syndrome, bicuspid aortic valve, and Ehlers-Danlos syndrome) were excluded from the analyses. Individuals who declined to have their medical records used for research were also excluded. Patients with idiopathic aortitis constituted our case group. The cohort of patients serving as controls was drawn from patients who were undergoing ascending aortic aneurysm repair during the same period and who fulfilled the exclusion criteria and did not have giant cell or lymphoplasmacytic inflammation in the wall of the resected aorta. For each case, two control subjects matched on age (± 5 years) and year of surgery were randomly selected from the pool of all controls with non-inflammatory non-infectious aneurysm.

### Data collection

Age, gender, and race were abstracted from the patient medical records. Cardiovascular risk factors assessed included gender, presence of hypertension, diabetes mellitus (types I and II), hyperlipidemia, family history of aneurysms, and smoking. Presence of hypertension, hyperlipidemia, or diabetes mellitus at the time of surgery was identified in the medical record by ICD-9 (International Statistical Classification of Diseases and Related Health Problems, ninth revision) coding or by physician diagnosis or the patient provided information on medical and family history during their visits to the Mayo Clinic (Rochester, MN, USA). Family history of any aortic aneurysm was collected from clinical records or patient family history forms. Smoking history at the time of surgery was classified as never, current (within last 30 days), or former (quit more than 30 days ago). ESR and CRP values were recorded if they had been measured within a month pre-operatively.

### Statistical analysis

Descriptive statistics were used to summarize the data (mean, median, proportions, and so on). The association between case/control status and cardiovascular risk factors was examined by means of logistic regression models. Each cardiovascular risk factor was examined individually and after adjustment for gender. Analyses are reported as odds ratio (OR) with corresponding 95% confidence intervals (CIs). The Fisher exact test was used to analyze percentage of cases versus controls with pre-operative elevation in ESR and CRP. In all cases, two-tailed *P *values of less than 0.05 were used to denote statistical significance. For risk factors with a prevalence of 25% to 50%, the study had 80% power to detect an OR of 2.9. For risk factors with a lower prevalence (for example, 10%) or a higher prevalence (for example, 70%), this study had 80% power to detect an OR of 4.0. The study was approved by the Mayo Clinic Institutional Review Board (number 08-008786) and was conducted according to its guidelines.

## Results

### Subjects

We identified 75 cases of non-infectious aortitis from patients who had undergone surgical repair during the study period. Of these, 25 cases were excluded, including patients with a history of GCA/PMR (n = 15), inflammatory arthritis (n = 2), Takayasu arteritis and Crohn disease (n = 1 each), bicuspid aortic valve (n = 3), and Marfan syndrome (n = 1). Two additional patients, one with a history of thymoma and one mislabeled as having aortitis without evidence of inflammation in the surgical specimen, were excluded. The clinical features, imaging findings, and surgical outcomes of 43 of these 50 patients with idiopathic aortitis have been described previously [10,11]. The control group consisted of 100 patients matched on age and year of surgery. The mean age of cases (± standard deviation) was 71.6 ± 8.9 years and that of controls was 71 ± 8.9 years (*P *= 0.69).

### Risk factors

The prevalence of cardiovascular risk factors is summarized in Table 1. Female gender was a risk factor for development of idiopathic aortitis (OR 2.41, 95% CI 1.20 to 4.85; *P *= 0.014). To reduce the probability of selection bias, we then compared the gender distribution of the 100 controls (69% were male) with that of the remaining 659 unselected controls (71.5% were male) from the pool of all patients undergoing surgery for this indication. There was no difference in gender distribution between the selected and unselected controls (*P *= 0.61).

**Table 1:** Comparison of cardiovascular risk factors in cases with idiopathic aortitis and control patients with non-inflammatory ascending thoracic aortic aneurysms

	Cases (n = 50)	Controls (n = 100)	Odds ratio (95% CI)	Odds ratio (95% CI) adjusted for gender
Gender female, percentage	52	31	2.41 (1.20, 4.85)	
Hypertension, percentage	76	69	1.42 (0.66, 3.09)	1.27 (0.57, 2.81)
Hyperlipidemia, percentage	45	51	0.78 (0.39, 1.56)	0.84 (0.42, 1.69)
Diabetes mellitus, percentage	6	14	0.39 (0.11, 1.43)	0.44 (0.12, 1.64)
Smoking, percentage				
Current	18	6		
Former	52	62	3.37 (1.12, 10.08)^a^	3.24 (1.05, 9.96)
Never	30	32		
Family history of aortic aneurysms, percentage	15	10	1.61 (0.56, 4.63)	1.37 (0.46, 4.06)

^a^Current versus never or former smokers. CI, confidence interval.

The prevalences of hypertension, hyperlipidemia, and family history of aortic aneurysms were similar in cases and controls (Table 1). The prevalence of current smokers was higher in cases as compared with controls (OR 3.37, 95% CI 1.12 to 10.08; *P *= 0.02). There was no difference in prevalence of former or never smokers between the groups. A trend toward a lower prevalence of diabetes mellitus was seen in cases as compared with controls (OR 0.39, 95% CI 0.11 to 1.43; *P *= 0.14).

To evaluate whether gender differences between cases and controls were masking the differences in cardiovascular risk factors, we performed gender-adjusted analyses (Table 1). There was no difference in prevalence of hypertension (OR 1.27, 95% CI 0.57 to 2.81; *P *= 0.56), hyperlipidemia (OR 0.84, 95% CI 0.42 to 1.69; *P *= 0.63), or family history of aortic aneurysms (OR 1.37, 95% CI 0.46 to 4.06; *P *= 0.57). The prevalence of current smokers continued to be higher even after adjustment for gender (OR 3.24, 95% CI 1.05 to 9.96; *P *= 0.04). There was a trend toward a lower prevalence of diabetes mellitus in patients with idiopathic aortitis (OR 0.44, 95% CI 0.12 to 1.64; *P *= 0.22).

### Acute-phase reactants

The pre-operative ESR and CRP measurements in the cases and controls are presented in Table 2. ESR was determined in 13 cases and 22 controls. The median values were 20 mm/hour in cases and 9 mm/hour in controls. Among those tested, a higher proportion of cases had an elevated ESR as compared with controls (38% versus 9%; *P *= 0.075). The median level of CRP was higher in cases among patients in whom the test was performed (n = 8, CRP = 12 mg/L) versus controls (n = 6, CRP = 3 mg/L; *P *= 0.010). A significantly higher proportion of cases had an elevated CRP as compared with controls (62% versus none; *P *= 0.031).

**Table 2:** Pre-operative erythrocyte sedimentation rate and C-reactive protein in cases with idiopathic aortitis compared with control patients with non-inflammatory aortic aneurysms

	Cases	Controls	*P *value
Pre-operative ESR			
Number tested	13	22	
Pre-operative ESR, mm/hour			
Mean ± SD	20.5 ± 11.6	11.3 ± 11.1	0.024
Median (range)	20 (2–40)	9 (0–40)	
Subjects with elevated ESR	38%	9%	0.075
Pre-operative CRP			
Number tested	5	6	
Pre-operative CRP, mg/L			
Mean ± SD	35 ± 50	3 ± 0.7	0.010
Median (range)	12 (2.5–119)	3 (1.2–3.2)	
Subjects with elevated CRP, percentage	60%	0%	0.031

CRP, C-reactive protein; ESR, erythrocyte sedimentation rate; SD, standard deviation.

## Discussion

To our knowledge, this is the first study to identify risk factors for development of idiopathic aortitis. Factors independently associated with an increased risk for idiopathic aortitis discovered at the time of surgical thoracic aneurysm repair in this study were female gender and active smoking. We did not find any difference in the prevalence of hypertension, hyperlipidemia, or family history of any aortic aneurysm. We found a trend, though not statistically significant, toward a lower prevalence of diabetes mellitus in cases versus controls.

The mechanism by which female gender predisposes to inflammation in the thoracic aorta is not known. The development of disease in older post-menopausal females suggests a role of sex hormones. Human aortic matrix is composed of collagen, which plays a role in load bearing, and elastin, which conveys elasticity to the aorta. Sex hormones play an important role in aortic wall compliance by regulating the elastin/collagen activity [12-14]. 17-β-Estradiol increases the elastin/collagen ratio, reflecting an increase in distensibility of aorta and consequently lower systolic blood pressure [15]. In animal models, oophorectomy increases collagen synthesis and decreases aortic distensibility [16]. Whether these hormones interact with immunologic and proteolytic systems to modulate inflammation in the stiff non-compliant older aorta merits further study.

We also found active smoking to be associated with an increased risk of idiopathic aortitis. The lack of any association with former or never smoking status indicates an acute but not cumulative effect. Smoking is an important risk factor for many rheumatic diseases like lupus and increases the risk of seropositivity in rheumatoid arthritis patients, especially those who are positive for shared epitope [17,18]. Smoking is the single most important factor for initiation and rapid growth of abdominal aortic aneurysms, and more patients with inflammatory abdominal aortic aneurysms tend to be smokers [19-27]. Smoking plays important roles in mediating atherosclerosis, elastolytic response, and potentiating inflammation [28-30]. It affects key proteolytic enzymes like MMPs, elastases, cysteine proteases, and lipoxygenases that are important for extracellular matrix degradation and aneurysm formation [31,32]. Exposure of endothelial cells to cigarette smoke increases MMP-1, MMP-8, and MMP-9 levels [33]. High serum levels of MMP-9 are found in moderate-diameter abdominal aortic aneurysms [34]. The importance of these processes is underscored by the fact that diabetic patients, in spite of their increased risk for atherosclerotic vascular disease, are at lower risk of abdominal aortic aneurysm [35,36]. Incubation of monocytes with glycated type 1 collagen matrices reduced the secretion of MMP-2, MMP-9, and IL-6 [37]. This may be one mechanism explaining why aneurysmal growth rate is slower in diabetic patients.

Smoking may also interact in complex mechanisms with immune response genes like human leukocyte antigen (HLA) to mediate vascular inflammation in predisposed individuals. In patients with inflammatory abdominal aortic aneurysms, active smoking and female gender were associated with high-grade tissue inflammation [21]. HLA-DR B1*01 has been reported to be protective and HLA-DR B1*02 and HLA-DR B1*04 (DR4, 0401 allele) were significantly associated with increased risk of tissue inflammation [21,38].

Though tested in only a subset of patients in our study, pre-operative ESR and CRP were elevated in a higher proportion of cases than controls. However, these elevations were much lower when compared with temporal arteritis patients with aortitis whose ESR levels range from 82 to 101 mm/hour [4-6]. It is unclear whether these markers should be determined pre-operatively in all patients undergoing aortic aneurysm repair or what the clinical consequence of elevated markers of inflammation should be in terms of potential therapy and follow-up.

The strength of our study is rigorous case definition with availability of histopathology in cases and controls. The case control design allowed us to study multiple risk factors for this very rare condition. The identification of risk factors is important for therapeutic intervention. Smoking cessation may slow down the inflammatory process and consequent growth of aneurysm [39]. Advances in molecular pathogenesis will pave the way for future therapies. Due to their anti-oxidant property, angiotensin-converting enzyme inhibitors have been reported to normalize impaired bradykinin-mediated endothelium-dependent venodilatation in smokers [40], and inhibition of MMP-9 by doxycycline was useful in preventing aneurysm growth [41,42].

The potential weaknesses of the study include the inclusion of surgical cases only. There may be a spectrum of disease, with mild disease not coming to medical attention. Inclusion of only cases with surgical specimens as the standard for evaluating inflammation was chosen to increase the internal validity of the study. Cases were selected from a large tertiary care referral center, perhaps introducing a potential bias for more severe disease. We have not analyzed the risk factors according to the histopathologic subsets of giant cell or lymphoplasmacytic inflammation; however, data from our retrospective cohort did not find any meaningful correlation between clinical features and histopathology due to small numbers (manuscript in preparation).

## Conclusion

Female gender and active smoking are risk factors for development of idiopathic aortitis. Future studies are needed to evaluate the utility of smoking cessation, the role of measurement of inflammatory markers, and medical treatment strategies on disease progress and outcome.

## Abbreviations

CI: confidence interval; CRP: C-reactive protein; ESR: erythrocyte sedimentation rate; GCA: giant cell arteritis; HLA: human leukocyte antigen; MMP: matrix metalloproteinase; OR: odds ratio; PMR: polymyalgia rheumatica.

## Competing interests

The authors declare that they have no competing interests.

## Authors' contributions

VRC conceived of the study and participated in study design, data acquisition, data interpretation, and manuscript preparation. CSC carried out statistical analysis. KPL, CJM, KJW, and ELM participated in study design and data interpretation. DVM carried out the pathologic review of aortic specimens. All authors read and approved the final manuscript.
